# Functions of CD1d-Restricted Invariant Natural Killer T Cells in Antimicrobial Immunity and Potential Applications for Infection Control

**DOI:** 10.3389/fimmu.2018.01266

**Published:** 2018-06-06

**Authors:** Yuki Kinjo, Shogo Takatsuka, Naoki Kitano, Shun Kawakubo, Masahiro Abe, Keigo Ueno, Yoshitsugu Miyazaki

**Affiliations:** Department of Chemotherapy and Mycoses, National Institute of Infectious Diseases, Tokyo, Japan

**Keywords:** invariant natural killer T cell, CD1d, glycolipid, adjuvant activity, microbial infection

## Abstract

CD1d-restricted invariant natural killer T (*i*NKT) cells are innate-type lymphocytes that express a T-cell receptor (TCR) containing an invariant α chain encoded by the *Vα14* gene in mice and *Vα24* gene in humans. These *i*NKT cells recognize endogenous, microbial, and synthetic glycolipid antigens presented by the major histocompatibility complex (MHC) class I-like molecule CD1d. Upon TCR stimulation by glycolipid antigens, *i*NKT cells rapidly produce large amounts of cytokines, including interferon-γ (IFNγ) and interleukin-4 (IL-4). Activated *i*NKT cells contribute to host protection against a broad spectrum of microbial pathogens, and glycolipid-mediated stimulation of *i*NKT cells ameliorates many microbial infections by augmenting innate and acquired immunity. In some cases, however, antigen-activated *i*NKT cells exacerbate microbial infections by promoting pathogenic inflammation. Therefore, it is important to identify appropriate microbial targets for the application of *i*NKT cell activation as a treatment or vaccine adjuvant. Many studies have found that *i*NKT cell activation induces potent adjuvant activities promoting protective vaccine effects. In this review, we summarize the functions of CD1d-restricted *i*NKT cells in immune responses against microbial pathogens and describe the potential applications of glycolipid-mediated *i*NKT cell activation for preventing and controlling microbial infections.

## Introduction

Natural killer T (NKT) cells are innate-type lymphocytes that recognize glycolipid antigens presented by the MHC class I-like molecule CD1d (1–7). The CD1d molecule is critical for both thymic development and the effector functions of NKT cells (1–7). These CD1d-restricted NKT cells are classified into two subpopulations, invariant NKT (*i*NKT) cells or type I NKT cells and type II NKT cells. In this review, we focus on *i*NKT cell functions in immunity and potential therapeutic applications, while the features and functions of type II NKT cells are summarized in several recent reviews (8, 9). The *i*NKT cell subtype expresses an invariant T-cell receptor (TCR) α chain encoded by the *V*α*14-J*α*18* gene in mice and *V*α*24-J*α*18* gene in humans. This TCRα chain is paired with a restricted repertoire of TCRα chains, such as Vβ8, 7, and 2 in mice and Vβ11 in humans (1–7). Upon TCR stimulation by microbe-derived glycolipid antigens or the potent synthetic lipid antigen α-galactosylceramide (α-GalCer), *i*NKT cells rapidly activate and produce large amounts of cytokines, including interferon-γ (IFNγ), interleukin-2, IL-4, IL-13, and IL-17A, and stimulate innate immune responses by activating antigen-presenting cells (APCs) and NK cells (1–6). *i*NKT cells activated by glycolipid antigens not only produce cytokines but also express CD40 ligand (CD40L), which stimulates the maturation of APCs, such as dendritic cells (DCs), leading to the augmentation of acquired immune responses (1–6). Through these unique signaling functions, CD1d-restricted *i*NKT cells participate in both innate and acquired immune responses against a variety of microbial pathogens, including bacteria, fungi, viruses, and protozoan parasites (10–15). In this review, we summarize the contributions of CD1d-restricted *i*NKT cells to immune responses against microbial pathogens by focusing on selected microbial infections. We also describe the potential applications of glycolipid-mediated *i*NKT cell activation for the development of new therapies and vaccines against infectious diseases.

## *i*NKT Cells Contribute to Innate Immune Responses Against Microbial Pathogens

Invariant natural killer T cells participate in the early phase of the immune response against various microbes through recognition of microbial components and stimulation of innate immune cells (10–15). Following infection by *Aspergillus fumigatus*, a major cause of invasive fungal infection in immunocompromised patients, *i*NKT cells produce IFNγ in response to recognition of endogenous antigens presented by CD1d, while APCs such as DCs release IL-12 in response to stimulation by β-glucan, resulting in the promotion of fungal clearance (16). Conversely, CD1d-deficient mice that lack *i*NKT cells exhibit delayed fungal clearance following infection by *A. fumigatus* (16).

*Streptococcus pneumoniae* (Pneumococcus) is the major cause of community-acquired pneumonia and meningitis, and is responsible for more than one million deaths annually. In the early phase of pneumococcal infection, *i*NKT cells contribute to neutrophil recruitment and bacterial clearance in the lungs through the release of neutrophil-recruiting cytokines such as tumor necrosis factor (TNF) and macrophage inflammatory protein-2 (17). Cell transfer experiments suggest that IFNγ produced by *i*NKT cells is essential for neutrophil recruitment (18). Other studies have reported that the *i*NKT cell response to Pneumococcus involves recognition of pneumococcal glycolipids (19, 20) and production of cytokines including IFNγ. The production of these cytokines is enhanced by IL-12 released from APCs stimulated by toll-like receptor (TLR) ligands of Pneumococcus (21).

CXCR6^+^ NKT cells, which consist mainly of *i*NKT cells, patrol liver sinusoids for signs of bacterial infection (22). When the TCR is stimulated by αGalCer, an anti-CD3 antibody, or bacterial glycosphingolipid, these CXCR6^+^ NKT cells stop crawling (22, 23). These observations suggest that *i*NKT cells play a major role in liver surveillance and arrest when stimulated. It has been demonstrated that Kupffer cells in liver sinusoids capture intravenously injected *Borrelia burgdorferi*, a causative bacterium of the inflammatory disorder Lyme disease (24, 25). *i*NKT cells then accumulate around *Borrelia*-ingested Kupffer cells and form clusters, a response dependent on the stimulation of the cytokine receptor CXCR3 (26). *Borrelia*-ingested Kupffer cells express CD1d and can, therefore, activate *i*NKT cells (26). *i*NKT cells have been shown to recognize a *B. burgdorferi* glycolipid presented by CD1d (27, 28), and the ensuing activation contributes to bacterial clearance and prevention of joint and heart inflammation (24, 25). Consistent with these observations, mice deficient in *i*NKT cells or depleted of Kupffer cells exhibited bacterial dissemination to bladder, joints, and heart (26). These results indicate that *i*NKT cells contribute to the immune response against *B. burgdorferi* during the early phase of infection by recognizing bacterial glycolipids presented by Kupffer cells in liver sinusoids, thereby preventing bacterial dissemination to other tissues.

Invariant natural killer T cells also participate in host protection against post-stroke bacterial infection, a major cause of stroke-related death. In mice, the number of crawling or CD69-expressing *i*NKT cells rapidly declined following transient middle cerebral artery occlusion, while the number of *i*NKT cells producing IL-10 (but not IFNγ or IL-4) increased (29). At 24 h after stroke, mice exhibited systemic infection as evidenced by the detection of endogenous bacteria in multiple organs and tissues (29). In contrast, the activation of *i*NKT cells by α-GalCer induced bacterial clearance from these organs and tissues. Furthermore, stroke-induced bacterial infection was prevented by the administration of propranolol, a nonspecific β-adrenergic receptor blocker, or by 6-hidroxydopamine, a neurotoxin that depletes peripheral neuronal terminals of noradrenaline, through recovery of *i*NKT cell functions such as crawling and IFNγ production and by shifting to a Th1-dominant response (29). Intriguingly, protection from bacterial infection by propranolol has not been observed in CD1d-deficient mice that lack *i*NKT cells. These results imply that stroke-associated infection is mediated by the suppression of *i*NKT cell function.

Collectively, these results indicate that *i*NKT cells play an important role in host defense against the early phase of microbial infection through the recognition of microbial glycolipids and stimulation of innate immune cells.

## Mechanisms of *i*NKT Cell Responses Against Microbial Pathogens

Previous studies have identified at least three mechanisms that trigger *i*NKT cell response to microbial pathogens: microbial glycolipid-mediated TCR activation, endogenous antigen-mediated weak TCR stimulation with concomitant inflammatory cytokine-mediated stimulation, and activation solely by inflammatory cytokines (2, 10, 12, 15).

Several microbial lipid antigens have been identified that activate *i*NKT cells through CD1d presentation to the TCR. For example, mouse and human *i*NKT cells recognize α-linked glycosphingolipids (GSLs) containing either a galacturonic acid or a glucuronic acid derived from commensal *Sphingomonas* species of the intestine (30–32). The structures of these glycolipids are very similar to α-GalCer, but with subtle differences such as the carbohydrate moiety and a shorter C14 acyl chain replacing the C26 acyl chain of α-GalCer (30, 31, 33). In addition to GSLs, *i*NKT cells also recognize glycerol-containing glycolipids. *B. burgdorferi* expresses a diacylglycerol containing α-linked galactose called *B. burgdorferi* glycolipid-II (BbGL-II). A BbGL-II isoform containing a palmitic acid (C16:0) and an oleic acid (C18:1) potently stimulated mouse *i*NKT cells (27, 28). Human *i*NKT cells respond more strongly to BbGL-II isoforms containing fatty acids with greater unsaturation, such as oleic acid (C18:1) and linoleic acid (C18:2) (27, 28). *Streptococcus pneumoniae* express an α-linked diacylglycerol containing a glucose (Glc-DAG). The Glc-DAG containing a palmitic acid (C16:0) and a vaccenic acid (C18:1) is recognized by mouse and human *i*NKT cells (19). These *Sphingomonas, B. burgdorferi*, and *S. pneumoniae* glycolipids act as antigens that stimulate mouse and human *i*NKT cell TCRs and induce cytokine release. One intriguing question is how the *i*NKT cell TCR with an invariant α chain recognize different antigens. Recent structural analyses of the *i*NKT cell TCR−glycolipid−CD1d ternary complex revealed that *i*NKT cell TCR induces conformational changes to both the bacterial glycolipid antigen and CD1d, thereby allowing the recognition of different glycolipid antigens by a conserved binding orientation (20, 33).

Activation of *i*NKT cells by combined endogenous antigen-mediated weak TCR stimulation and inflammatory cytokine-mediated stimulation (2, 10, 12) is exemplified by *Salmonella typhimurium*, a Gram-negative bacterium expressing lipopolysaccharide (LPS). *S. typhimurium* has been shown to stimulate IFNγ release from *i*NKT cells despite the absence of a recognized glycolipid antigen (34). The activation of *i*NKT cells by *S. typhimurium* is mediated by IL-12 released from APCs stimulated by LPS through TLR4 and myeloid differentiation primary response 88 signaling (34). In addition, *i*NKT cell activation is partially dependent on CD1d (34), suggesting that *i*NKT cell activation during *S. typhimurium* infection requires a combination of weak TCR stimulation by an endogenous antigen and stimulation by inflammatory cytokines released by APCs in response to *S. typhimurium*.

In other cases, *i*NKT cells are activated solely by inflammatory cytokines (10, 12, 15). In the early phase of murine cytomegalovirus (MCMV) infection, a substantial number of *i*NKT cells produce IFNγ (35). However, this MCMV-associated cytokine production is independent of CD1d, but highly dependent on IL-12 and partially dependent on type I IFN (35). *i*NKT cells also amplify IFNγ release from NK cells and contribute to host protection against MCMV infection (35). In Nur77^gfp^ reporter mice harboring T cells that express green fluorescent protein (GFP) upon antigen-mediated TCR stimulation, but not inflammatory cytokines, MCMV infection induced IFNγ production by *i*NKT cells without GFP expression (36). Collectively, these results show that the *i*NKT cell response to MCMV is independent of TCR stimulation but dependent on inflammatory cytokines. In contrast to MCMV, *S. pneumoniae* and *Sphingomonas paucimobilis* induced the expression of GFP and IFNγ in *i*NKT cells, indicating that these species activate *i*NKT cells through TCR stimulation (36). Alternatively, *S. typhimurium* and LPS did not induce GFP expression by *i*NKT cells, although these cells did produce IFNγ (36). These results suggest that inflammatory signals play an important role in *i*NKT cell activation in response to microbes that do not possess glycolipid antigens, greatly expanding the spectrum of *i*NKT cell-activating pathogens.

## *i*NKT Cells Contribute to Acquired Immune Responses Against Microbial Pathogens

*Cryptococcus neoformans* is a fungal pathogen that causes pulmonary infection and can also disseminate to the central nervous system and cause meningitis, especially in immunocompromised individuals such as those with acquired immune deficiency syndrome. Following pulmonary infection of mice with *C. neoformans, i*NKT cells accumulated in the lungs, a response dependent on monocyte chemoattractant protein-1 (37). Jα18-deficient mice lacking *i*NKT cells exhibited delayed fungal clearance due to a weak Th1 response, normally a key immune response against *C. neoformans* infection (37). These results suggest that *i*NKT cells contribute to protection against cryptococcal infection through the stimulation of Th1 response. It was subsequently reported, however, that Jα18-deficient mice also exhibit defects in the rearrangement of Jα segments upstream of Jα18 (38). Therefore, the results obtained in Jα18-deficient mice may not be solely due to *i*NKT cell deficiency, especially under conditions involving the adaptive immune response.

A recent study has revealed an important role of *i*NKT cells in the initial formation of germinal centers during influenza infection. *i*NKT cells are a major source of early IL-4 release during infection, which is essential for the induction of germinal center B cells and ensuing IgG1 production (39). This enhanced IL-4 release is triggered by CD1d and IL-18 stimulation from CD169^+^ macrophages (39). Furthermore, the transcriptomic analysis of lymph nodes in Zika virus-infected macaque monkeys revealed that IL-4 and NKT cell signatures, but not the Tfh cell signature, was strongly correlated with neutralizing antibody titer in the early phase of infection (39). These results suggest that *i*NKT cells promote initial germinal center formation and IgG production during the early stage of viral infection through macrophage-induced IL-4 release.

Taken together, these results highlight the importance of *i*NKT cells in host defense against various microbial infections through the stimulation of both innate and acquired immunity.

## Glycolipid-Mediated Activation of *i*NKT Cells Enhances Antimicrobial Immunity

As discussed, *i*NKT cells contribute to neutrophil recruitment during pneumococcal infection (17, 18). For instance, the activation of *i*NKT cells by α-GalCer promoted bacterial clearance through the recruitment of neutrophils and protected mice from lethal pneumococcal infection (17). Respiratory DCs, especially CD103^+^ DCs, promote *i*NKT cell activation through the release of IFNγ and IL-17A, key cytokines conferring protection against pneumococcal infection (40). Stimulation of *i*NKT cells by α-GalCer also induces macrophage activation. During lung infection by *Pseudomonas aeruginosa*, α-GalCer treatment increased IFNγ and TNF in bronchoalveolar lavage fluid and the phagocytosis of bacteria by alveolar macrophages, resulting in rapid recovery from pneumonia (41). It has also been shown that α-GalCer treatment significantly inhibits malaria infection at the liver stage, but not at the blood stage, in an IFNγ-dependent manner (42). Alpha-C-galactosylceramide (α-C-GalCer), a C-glycoside analog of α-GalCer, induces longer IFNγ production and lower IL-4 production than α-GalCer (43), and α-C-GalCer has been shown to exhibit superior antimicrobial efficacy during the liver stage of malaria infection compared with α-GalCer (43). This superior effect of α-C-GalCer is dependent on IL-12, which is necessary for IFNγ production by NK cells, a major source of IFNγ. These results indicate that glycolipid-mediated *i*NKT cell activation enhances innate immune responses, resulting in a greater control of microbial infections at the early phase.

Glycolipid-activated *i*NKT cells augment the induction of effector CD4T cells and CD8T cells through the activation of APCs such as DCs. During *Chlamydophila pneumoniae* infection, α-GalCer-activated *i*NKT cells upregulated CD40 expression and IL-12 production by DCs, leading to the expansion of IFNγ-producing CD4T cells and IFNγ-producing CD8T cells and ultimately decreasing the bacterial burden in lungs (44, 45). It has also been shown that α-GalCer treatment enhances the Th1 response and fungal clearance during *C. neoformans* infection in an IFNγ-dependent manner (46). In the absence of IL-18, the increased IFNγ production and inhibition of fungal growth induced by α-GalCer were further enhanced through a greater production of IL-12 and IL-4 (47). Alpha-GalCer treatment also increases the memory CD4T cell pool size and alters the function of memory Th2 cells for increased IFNγ production (48). Further, α-GalCer treatment promotes the differentiation of central memory CD8T cells. During MCMV infection, α-GalCer treatment rapidly induced IFNγ and IL-4 production and decreased viral titers in spleen and liver (49). These α-GalCer-treated mice also exhibited greater numbers of MCMV antigen-specific central memory CD8T cells (49). These results suggest that glycolipid-mediated *i*NKT cell activation may be an effective strategy to augment the induction of effector and memory CD4T cells and CD8T cells that contribute to host protection against microbial infections.

## *i*NKT Cells Contribute to the Pathogenesis of Some Microbial Infections

In contrast to these documented benefits, *i*NKT cells play a detrimental role against the host during certain microbial infections by the induction or augmentation of inflammation, which results in the exacerbation of infection or causes severe acute or chronic inflammatory diseases (12, 13). *Candida* species colonize the skin and gastrointestinal and genitourinary mucosal surfaces and are a major cause of bloodstream infections among inpatients, with mortality rates from candidemia and invasive candida infections as high as 30−40% (50, 51). *i*NKT cells contribute to the pathogenesis of *C. albicans* infection, the most frequent *Candida* species. Following systemic *C. albicans* infection, Jα18-deficient mice lacking *i*NKT cells exhibited a higher survival rate and a lower fungal burden in various organs than wild-type (WT) mice because of the increased accumulation of macrophages and neutrophils in the peritoneal cavity (52). Consistent with the amelioration of infection by *i*NKT cell depletion, IL-10 levels were lower and IL-12p40 levels were higher in the serum of *C. albicans*-infected Jα18-deficient mice than infected WT mice. Conversely, NKT cell transfer exacerbated *C. albicans* infection in Jα18-deficient mice concomitant with reduced accumulation of macrophages and neutrophils (52). Furthermore, IL-10 treatment exacerbated *C. albicans* infection in Jα18-deficient mice, and transfer of IL-10-deficient NKT cells into Jα18-deficient mice significantly increased survival following *C. albicans* infection compared to the transfer of WT NKT cells (52). However, another study found no difference in susceptibility to *C. albicans* infection between Jα18-deficient and WT mice (53). This discrepancy is probably because of the different *C. albicans* strains employed and distinct routes of infection. It should also be reiterated that the difference in infection response by Jα18-deficient mice may not be due to *i*NKT cell deficiency alone, as these mice also show deficits in the rearrangement of Jα segments upstream of Jα18 (38).

Alpha-GalCer-mediated *i*NKT cell activation also exacerbates *C. albicans* infection. Alpha-GalCer-treated mice exhibited higher fungal burden in kidneys, higher IL-6 levels in serum and kidneys, wider dissemination of fungi, and shorter survival than control-infected mice (54). The number of neutrophils, the main effector cells controlling *C. albicans* infection, was significantly decreased in *C. albicans* infected and α-GalCer-treated mice, and this difference was IFNγ-dependent (54). It is thought that some bacterial species can disseminate to blood from the intestine in immunocompromised patients and activate *i*NKT cells. Furthermore, this mode of *i*NKT cell activation may exacerbate certain infections. Mice pre-infected with *Sphingomonas* bacteria, which are commensal and possess glycolipid antigens for *i*NKT cells (30–32), prior to *C. albicans* exposure exhibited enhanced IFNγ-dependent *i*NKT cell activation, increased production of inflammatory cytokines, and greater fungal burden (54). Collectively, these results indicate that *i*NKT cells participate in the pathogenesis of *C. albicans* infection and that *i*NKT cell activation by glycolipid antigens or bacterial infection can exacerbate *C. albicans* infection.

## Glycolipid-Activated *i*NKT Cells Exhibit Effective Adjuvant Activities to Prevent Microbial Infections

Many studies have demonstrated the potential adjuvant activities of glycolipid-activated *i*NKT cells for protection against microbial infections (11, 12, 14). For instance, immunization with malarial antigens and α-GalCer inhibited the liver stage of malaria and prevented parasitemia more effectively than malarial antigen alone. Immunization with malarial antigens and α-GalCer also increased the number of IFNγ-producing antigen-specific CD8T cells, major effector cells controlling the liver stage of malaria infection (55). Mice sublingually immunized with the *Mycobacterium tuberculosis* antigens Ag85B and ESAT-6 together with α-GalCer exhibited stronger antigen-specific CD4T- and CD8T-cell responses than mice immunized with Ag85B and ESAT-6 alone, and resulted in a significantly lower organ bacterial burden (56). Immunization with bacillus Calmette–Guérin (BCG)-incorporated α-GalCer or α-C-GalCer, an analog with a C-glycoside, induced a greater number of antigen-specific IFNγ-producing CD8T cells than unmodified BCG through increased maturation of DCs by *i*NKT cells (57). Mice immunized with glycolipid-incorporated BCG also exhibited reduced bacterial loads in lungs and spleen compared with mice receiving unmodified BCG immunization (57). These vaccine effects were more evident with α-C-GalCer than α-GalCer, probably due to the lower IL-4 production and prolonged IL-12 production induced by α-C-GalCer compared to α-GalCer (43).

Glycolipid-mediated *i*NKT cell activation also augments antibody production by B cells (58, 59). Intranasal administration of α-GalCer and influenza hemagglutinin (HA) vaccine or formalin-inactivated whole-virion vaccine induced higher titers of mucosal IgA and systemic IgG compared to influenza vaccine alone (60–62). Co-administration of α-GalCer and influenza vaccine also protected mice from lethal influenza virus infection, including H5N1 influenza virus infection (62), through enhanced viral clearance (60–62). Glycolipid-mediated *i*NKT cell activation also has adjuvant activity in swine. Indeed, α-GalCer showed excellent adjuvant activity with UV-inactivated influenza virus for increasing virus-specific antibody titers and IFNγ-producing cells in swine, and this immunization strategy protected against pandemic H1N1 influenza infection (63).

Although it is well known that follicular helper T (T_FH_) cells play a critical role in the stimulation of germinal center B cells for high-affinity antibody production, as well as differentiation of memory B cells and long-lived plasma cells, recent studies have demonstrated that follicular helper NKT (NKT_FH_) cells contribute to augmented IgG antibody production by vaccines containing an *i*NKT cell glycolipid antigen (59, 64–67). Immunization of mice with liposomes containing pneumococcal capsular polysaccharide (CPS) and PBS57, an α-GalCer analog, or CPS-α-GalCer conjugate vaccine induced NKT_FH_ cells expressing PD-1 and CXCR5 or PD-1 and ICOS (66, 67). Mice treated with these vaccines showed an enhanced IgG1 production, indicating that cognate B cells are activated by T cells. Intriguingly, IgG1 production was dependent on CD1d expression by B cells and DCs, indicating that IgG1 production is induced by the cognate interaction of *i*NKT cells and B cells (66). These vaccines containing pneumococcal CPS and a glycolipid induced germinal center formation and CPS-specific memory B cells and long-lived plasma cells, which provided long-term protection against pneumococcal infection (67).

**Figure 1:**
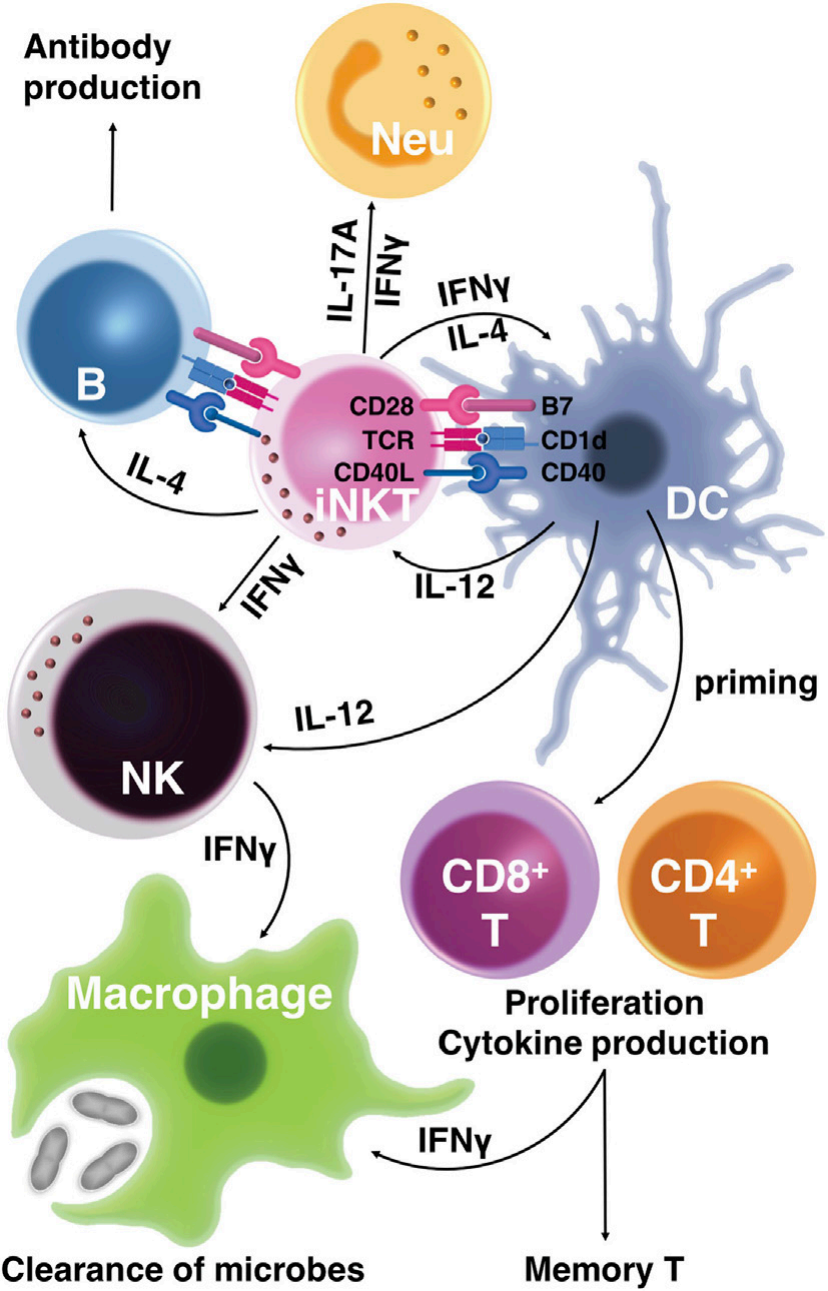
Activation of CD1d-restricted invariant natural killer T (*i*NKT) cells augments both innate and acquired immunity to control microbial infection. The T-cell receptor (TCR) of *i*NKT cells recognizes glycolipid antigens presented by CD1d on antigen-presenting cells (APCs). In response, activated *i*NKT cells produce cytokines, including interferon-γ (IFNγ), interleukin-4 (IL-4), and IL-17A, that stimulate innate immune responses such as neutrophil (Neu) recruitment. Glycolipid-activated *i*NKT cells also express CD40 ligand (CD40L), which promotes APC maturation. *i*NKT cells provide cognate help to B cells to promote antibody production when glycolipid-conjugated antigens are presented by B cells. Through cytokine release and CD40L–CD40 interaction, *i*NKT cells stimulate dendritic cells (DCs), triggering DC production of cytokines such as IL-12. These DC-derived cytokines stimulate IFNγ production by *i*NKT cells, which in turn enhances microbial clearance by stimulating macrophages. Activated *i*NKT cells also induce maturation of DCs that prime IFNγ-producing effector CD4T and CD8T cells, resulting in the clearance of microbes. The mature DCs induced by activated *i*NKT cells enhance the differentiation not only of effector T cells but also of memory T cells, conferring long-term protection against microbial infection.

Collectively, glycolipid-mediated *i*NKT cell activation provides excellent adjuvant activities for inducing effector T-cell responses, promoting high-affinity antibody production by B cells, and for augmenting memory T- and B-cell responses (Figure 1).

## Concluding Remarks

Due to space limitations, this review focused on only a few selected studies of *i*NKT cell responses to microbes. However, numerous studies have demonstrated that CD1d-resticted *i*NKT cells contribute to immune responses against a broad spectrum of pathogenic microbes despite constituting only a small fraction of the leukocyte population. Due to the capacity for rapidly producing large quantities of cytokines in response to TCR stimulation, glycolipid-activated *i*NKT cells can augment both innate and acquired immunity, thereby providing protection against disparate microbial pathogens. However, in some cases, glycolipid-mediated *i*NKT cell activation may contribute to the pathogenesis of infection by exacerbating inflammation. Therefore, it is critical to distinguish which microbial targets are suppressed by *i*NKT cell activation for treatment or vaccine development. Considering the accumulating evidence for excellent adjuvant activities of glycolipid-mediated *i*NKT cell activation and the strong evolutionary conservation of *i*NKT cell responses to glycolipid antigens among experimental animals (usually mice) and humans (10–12), the inclusion of glycolipids inducing *i*NKT cell activation in vaccination regimens may be an effective strategy to prevent and control microbial infections in humans. It should be noted, however, that human and mouse *i*NKT cell responses to some glycolipids may differ as previously demonstrated by two C-glycoside analogs of α-GalCer (68) and *Borrelia* glycolipids (27). Therefore, careful consideration is needed when choosing a glycolipid antigen for clinical application of glycolipid-mediated *i*NKT cell activation.

## Author Contributions

All authors contributed to this work and approved submission for publication.

## Conflict of Interest Statement

The authors declare that the research was conducted in the absence of any commercial or financial relationships that could be construed as a potential conflict of interest.
